# Identification of Tp0751 (Pallilysin) as a *Treponema pallidum* Vascular Adhesin by Heterologous Expression in the Lyme disease Spirochete

**DOI:** 10.1038/s41598-017-01589-4

**Published:** 2017-05-08

**Authors:** Wei-Chien Andrew Kao, Helena Pětrošová, Rhodaba Ebady, Karen V. Lithgow, Pablo Rojas, Yang Zhang, Yae-Eun Kim, Yae-Ram Kim, Tanya Odisho, Nupur Gupta, Annette Moter, Caroline E. Cameron, Tara J. Moriarty

**Affiliations:** 1 Matrix Dynamics Group, Faculty of Dentistry, University of Toronto, Toronto, ON Canada; 2 Department of Biochemistry and Microbiology, University of Victoria, Victoria, BC Canada; 3 Charité University Medicine Berlin, Berlin, Germany; 4 Biofilmcenter, German Heart Center Berlin, Berlin, Germany; 5 Department of Laboratory Medicine and Pathobiology, Faculty of Medicine, University of Toronto, Toronto, ON Canada

## Abstract

*Treponema pallidum* subsp. *pallidum*, the causative agent of syphilis, is a highly invasive spirochete pathogen that uses the vasculature to disseminate throughout the body. Identification of bacterial factors promoting dissemination is crucial for syphilis vaccine development. An important step in dissemination is bacterial adhesion to blood vessel surfaces, a process mediated by bacterial proteins that can withstand forces imposed on adhesive bonds by blood flow (vascular adhesins). The study of *T*. *pallidum* vascular adhesins is hindered by the uncultivable nature of this pathogen. We overcame these limitations by expressing *T*. *pallidum* adhesin Tp0751 (pallilysin) in an adhesion-attenuated strain of the cultivable spirochete *Borrelia burgdorferi*. Under fluid shear stress representative of conditions in postcapillary venules, Tp0751 restored bacterial-vascular interactions to levels similar to those observed for infectious *B*. *burgdorferi* and a gain-of-function strain expressing *B*. *burgdorferi* vascular adhesin BBK32. The strength and stability of Tp0751- and BBK32-dependent endothelial interactions under physiological shear stress were similar, although the mechanisms stabilizing these interactions were distinct. Tp0751 expression also permitted bacteria to interact with postcapillary venules in live mice as effectively as BBK32-expressing strains. These results demonstrate that Tp0751 can function as a vascular adhesin.

## Introduction

*Treponema pallidum* subsp. *pallidum* is a bacterium from the spirochete family and the causative agent of syphilis. Syphilis is a sexually transmitted disease, which is often considered a disease of the past. However, it still represents a global health problem with 36 million cases and 11 million new infections annually^1^. Moreover, syphilis incidence has increased dramatically over the past two decades in Canada^2^, the United States^3^, China^4^, and in a number of European countries^5–7^. Given its high prevalence, syphilis has a substantial impact on global health. Syphilis infection in pregnancy has up to 80% morbidity and can result in stillbirth, neonatal death, and complications associated with congenital syphilis in newborns^8^. Active syphilis infection also significantly increases infectivity of HIV-positive patients and risk of HIV acquisition^9^, and modeling studies suggest that controlling syphilis will contribute to HIV prevention^10^. Since elimination of syphilis has not been achieved by antibiotic treatment, novel preventative approaches are needed to fight this disease.

To date, the molecular mechanisms of *T*. *pallidum* pathogenesis are still poorly understood. This is mainly because the organism is an obligate human pathogen that cannot be cultured continuously *in vitro* ^11^. The inability to culture *T*. *pallidum* has limited the use of many experimental approaches, including genetic manipulation to study the molecular pathogenesis of this bacterium. To overcome this issue, a heterologous gain-of-function system for expressing candidate *T*. *pallidum* virulence genes in related culturable organisms is needed. The *T*. *pallidum* protein Tp0751 has been successfully expressed in the culturable non-adherent spirochete *Treponema phagedenis*, where it restores the capacity for host cell adhesion under static conditions^12^. Recently, expression of a candidate *T*. *pallidum* protein (Tp0435) in the Lyme disease spirochete *Borrelia burgdorferi* has been used to establish its role in immune evasion and host cell adhesion^13^.

*T*. *pallidum* disseminates widely via the bloodstream to invade multiple tissues and organs and the interaction of *T*. *pallidum* with the vasculature is therefore a critical step of the infection process^14^. Dissemination allows the pathogen to spread from the initial site of infection to secondary target tissues including, but not limited to, the oral cavity^15^, kidney^16^, stomach^17^, spleen^18^, liver^18^, and central nervous system^19^. *T*. *pallidum* disseminates not only widely, but rapidly; *T*. *pallidum* can penetrate endothelial intracellular junctions^20–23^, and in a rabbit model enters the circulatory system within minutes of initial infection^24^. It also is one of the few pathogens able to cross both the blood-brain and placental barriers^11, 19, 25^. Identification of the mechanisms underlying the extraordinary invasiveness of this pathogen is a key to understanding and preventing disease progression.

In order to invade tissue barriers such as the endothelial lining of blood vessels, bacteria must first attach to host ligands associated with endothelial surfaces via bacterial adhesion proteins (adhesins)^26^. These interactions permit bacteria circulating at the speed of blood flow to slow down and transmigrate through the endothelial lining of blood vessels (extravasation). Interaction of circulating bacteria with endothelia and its associated extracellular matrix (ECM) is challenging under vascular shear stress conditions. The first step of this process, in which surface molecules on blood-borne bacteria interact with molecules on endothelial surfaces, is especially critical, since binding interactions must be sufficiently strong and stable to withstand the fluid shear stress (force) generated by blood flow over vessel walls. Circulating host cells such as leukocytes, for example, must slow and roll along endothelial surfaces before they can extravasate. Even in the vessels where shear stress is typically lowest (postcapillary venules: PCVs), leukocytes require specialized adhesion molecules with catch bond properties (bonds which strengthen as force increases) and/or elastic, bungee cord-like tethers, which anchor leukocytes to endothelial surfaces to stabilize interactions^27^. In the absence of such stabilizing mechanisms, leukocyte-endothelial interactions are very vulnerable to small changes in force, and leukocytes dissociate from endothelia at exponentially faster rates with small, linear increases in shear stress^28^.

Disseminating bacteria also require specialized adhesins and interaction mechanisms that can withstand the forces generated by blood flow, since such mechanisms are necessary for bacteria to slow down and extravasate through the endothelial lining of blood vessels. *B*. *burgdorferi*-endothelial interactions mediated by the adhesin BBK32 become more stable with increasing force under PCV shear stress conditions and thus exhibit catch bond properties, and both BBK32-dependent and BBK32-independent *B*. *burgdorferi*-endothelial interactions are stabilized by tethers^29^. BBK32-mediated catch bonds also depend on plasma fibronectin (pFn)^30^. In addition to BBK32, a number of other vascular adhesins have recently been identified in *B*. *burgdorferi* (BB0347, and decorin-binding proteins DbpA and DbpB)^31–33^ and in *Staphylococcus aureus* (von Willebrand factor-binding protein, vWbp, and fibronectin-binding protein A, FnBPA)^34, 35^. All of these adhesins, including BBK32, share the property of binding to glycosylated structures and/or extracellular matrix components of blood and endothelial cells, including von Willebrand factor and fibronectin (Fn)^33, 36–39^. Additional bacterial adhesins and virulence factors such as the *B*. *burgdorferi* adhesin P66^40^ are also likely to be important for transmigration of bacteria through endothelial layers after or during shear stress-resistant adhesion. Adhesion of *T*. *pallidum* to endothelial surfaces under physiological shear stress conditions has not been studied, and adhesins that are strong and stable enough to permit vascular interactions during dissemination of this pathogen have not yet been identified. Identification of adhesins that promote cardiovascular dissemination of *T*. *pallidum* will be important for vaccine development to prevent the establishment of disseminated infection.

Several *T*. *pallidum* proteins have been identified as candidate adhesins, as determined by binding of recombinant proteins to ECM components *in vitro* ^41–44^. One of these proteins, Tp0751 (pallilysin), has been localized to the *T*. *pallidum* surface^45^ and has been found to bind the host ECM components laminin^42^, fibrinogen^46^, fibronectin (Fn)^47^, and collagen^47^, which are important constituents of blood and endothelia. It has been predicted that Tp0751 promotes dissemination by attaching to host ECM components and by facilitating *T*. *pallidum* localization to cell junctions^45, 46, 48^. The Tp0751 crystal structure revealed that interactions of Tp0751 with ECM components are facilitated by its unique lipocalin domain^49^. A lipocalin domain that shares similar unique attributes to Tp0751 has been identified in the *Neisseria meningitidis* adhesin fHbp, which is a key component of the Meningococcal B vaccines^50^. Similar to the fHbp protein, Tp0751 represents a promising vaccine candidate, as vaccination with Tp0751 inhibits dissemination of *T*. *pallidum* within the rabbit host^51^.

Due to the propensity for Tp0751 to bind to host cell components found in blood and the vasculature and the ability of Tp0751 immunization to inhibit treponemal dissemination, in the present study we investigated whether Tp0751 can mediate vascular adhesion under the force conditions found in the vasculature. We heterologously expressed Tp0751 on the surface of a non-infectious adhesion-attenuated strain of *B*. *burgdorferi*, which was previously used as a gain-of-function model system to identify BBK32 as a vascular adhesin^31, 49^. We then evaluated whether *B*. *burgdorferi* expressing Tp0751 could interact with human endothelia under both static and physiological shear stress conditions, characterized the biophysical properties of these interactions (strength and stability), and determined if Tp0751 could function as a vascular adhesin in the PCVs of live mice. The methods we have used to characterize the role of Tp0751 in vascular adhesion provide a powerful new repertoire of approaches for studying *T*. *pallidum* pathogenesis, and will permit identification of other vascular adhesins and the underlying mechanisms of dissemination in this important, under-studied human pathogen.

## Results

### Adhesion of Tp0751-expressing *B*. *burgdorferi* to human endothelia under static conditions

**Figure 1:**
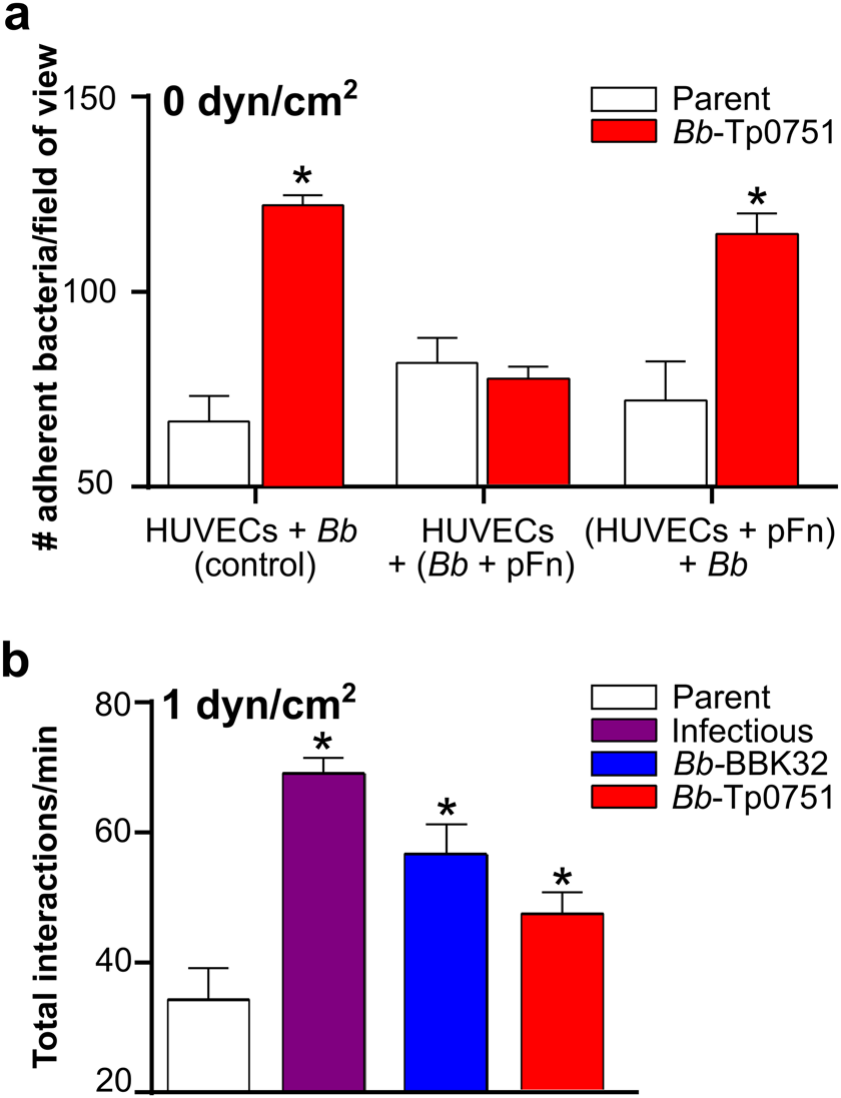
Interaction of Tp0751-expressing strains with human endothelia under static and shear stress conditions. (**a**) Mean ± SEM numbers of parent and Tp0751-expressing *B*. *burgdorferi* (*Bb*-Tp0751) adhered to primary human umbilical vein endothelial cell (HUVEC) monolayers after 12 h of co-incubation under static (0 dyn/cm^2^) conditions (HUVECs + *Bb*, control). HUVECs+ (*Bb* + pFn): binding of bacteria pre-incubated with plasma fibronectin (pFn) to HUVECs. (HUVECs + pFn) + *Bb*: binding of bacteria to HUVEC pre-incubated with pFn. N = 3 independent bacterial and endothelial cultures/strain analyzed in 2 experiments. (**b**) Mean ± SEM bacterial interactions per minute with HUVEC under typical postcapillary venule shear stress condition (1 dyn/cm^2^). Videos of representative flow chamber experiments are presented in Supplementary Videos S1–S3. Numbers of bacteria that paused and moved more slowly over endothelia under flow were manually counted in time-lapses acquired at 15 frames per second (fps). Strains: Negative control strain: BBK32-deficient non-infectious adhesion-attenuated B31-A-derived strain (Parent; GCB706: Supplementary Table S1). Positive control strains: Parent expressing *B*. *burgdorferi* vascular adhesin BBK32 (*Bb*-BBK32); BBK32-expressing B31-derived infectious strain (Infectious; GCB726: Supplementary Table S1). *Bb*-Tp0751: Parent expressing Tp0751. N = 8 independent cultures per strain analyzed in 3 experiments. Statistics: one-way Kruskal-Wallis ANOVA with Dunn’s post-test (**a**), one-way ANOVA with Holm-Sidak post-test (**b**). *Indicates p < 0.05 vs. parent within incubation condition (**a**) and vs. parent (**b**).

It has been hypothesized that Tp0751 contributes to vascular dissemination of *T*. *pallidum* due to the capacity of this adhesin to attach to ECM components located in close proximity to the vasculature^45–47^. To test this hypothesis, we cloned the full length Tp0751 gene fused to a C-terminal 3XFLAG tag into a non-infectious BBK32-deficient adhesion-attenuated green fluorescent protein (GFP)-expressing strain of *B*. *burgdorferi*, resulting in Tp0751 localization to the bacterial surface (strain *Bb*-Tp0751)^49^. Since virulent *T*. *pallidum* readily attaches to primary human umbilical vein endothelial cells (HUVEC)^22, 23, 52^, we then measured the ability of *Bb*-Tp0751 to adhere to monolayers of HUVEC in 12-hour co-incubations in the absence of fluid shear stress (Fig. 1). Under these static conditions, bacteria expressing Tp0751 adhered to endothelia significantly more efficiently than the adhesion-attenuated parent strain (Fig. 1a; “control”). Tp0751-dependent endothelial adhesion was inhibited by pre-incubation of bacteria with plasma fibronectin (pFn; the main source of fibronectin in blood) before addition to endothelia, but not by pre-incubation of endothelia with pFn before addition of bacteria (Fig. 1a). This result suggests that either Fn already present on endothelia was sufficient to mediate interactions, or that interactions may be occurring directly with endothelial surfaces. Tp0751 might therefore mediate attachment to endothelia in a specific fashion that may be partially dependent upon the presence of pFn.

### Interaction of Tp0751-expressing *B*. *burgdorferi* with human endothelia under postcapillary venule shear stress conditions

In the circulatory system, fluid shear stress created by blood flow over vessel walls has a profound impact on the interactions of circulating cells with vascular surfaces^27, 53^. We therefore investigated whether Tp0751 could support *B*. *burgdorferi* interactions with HUVEC monolayers in flow chambers at the typical shear stress encountered in PCVs (1 dyn/cm^2^), which is the site of most *B*. *burgdorferi*-vascular interactions^54^. Interactions were measured by manually counting numbers of bacteria that paused on endothelial surfaces but moved faster than 100 µm/s (tethered; tethered interactions), and numbers of bacteria which paused and moved more slowly than 100 µm/s (dragged; untethered interactions) along endothelial surfaces (Fig. 1b)^29, 31, 54^. Interactions were counted for the negative control parent and *Bb*-Tp0751, as well as parent-derived bacteria expressing C-terminally FLAG-tagged BBK32 (positive control: *Bb*-BBK32). We have previously shown that gain of function expression of untagged BBK32 in this parent strain restores PCV interactions to the level observed for infectious *B*. *burgdorferi* ^31, 55^. It was therefore not surprising that expression of FLAG-tagged BBK32 restored total interaction numbers to the same level observed for infectious bacteria (p > 0.05; Fig. 1b). The FLAG tag itself did not promote bacterial-endothelial interactions, as addition of the FLAG tag did not affect endothelial interaction numbers or velocities at 1 dyn/cm^2^ for a parent strain expressing RevB (p > 0.05; Supplementary Fig. S1), a *B*. *burgdorferi* adhesin which does not restore vascular interactions to the adhesion-attenuated parent^55^. Collectively, interaction numbers were similar for *Bb*-Tp0751 and *Bb*-BBK32 (p > 0.05; Fig. 1b), and were greater than for the parent strain (p < 0.05; Fig. 1b), suggesting that Tp0751 acts as an endothelial adhesin under physiological shear stress.

### Tp0751 stabilizes and slows *B*. *burgdorferi*-endothelial interactions under shear stress

**Figure 2:**
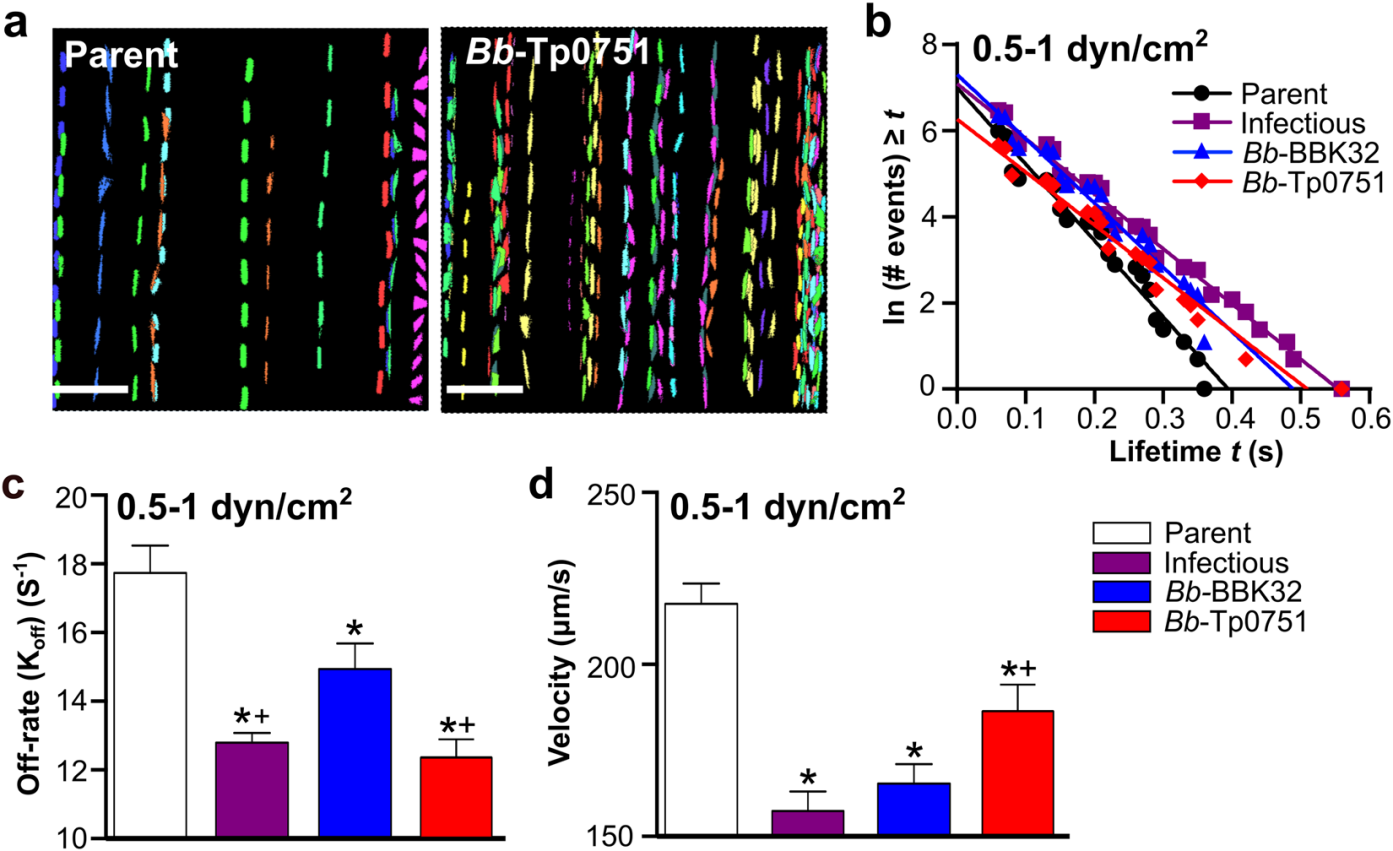
Bond dissociation rates and interaction velocities of Tp0751-expressing *B*. *burgdorferi*. *B*. *burgdorferi* interactions with HUVEC in flow chambers at 0.5 and 1 dyn/cm^2^ were captured by particle tracking of interaction trajectories (tracks) in time lapse sequences. (**a**) Sample time lapse projections depicting interaction tracks of individual bacteria (1 color/bacterium) captured at 1 dyn/cm^2^ over 2 min. Scale bars: 38 µm. (**b**) The linear regression of the log-transformed number of interactions versus the lifetime (seconds) of the spirochete for 4 representative tracks (1 track for each of the 4 strains). The negative slope of each regression was an estimate of the dissociation rate (K_off_) of the spirochete, as described in the Materials and Methods. (**c**) Mean ± SEM dissociation rates (K_off_/off-rates) for individual load-bearing adhesion complexes mediating bacterial-endothelial interactions under flow. (**d**) Velocities of interacting bacteria (mean ± 95% CI). In (**b**–**d**), bond dissociation rates and interaction velocities were calculated from interactions obtained at both 0.5 and 1 dyn/cm^2^. N = 4 and N = 8 independent flow chamber experiments for each strain at 0.5 and 1 dyn/cm^2^, respectively. Eight replicate experiments were conducted at 1 dyn/cm^2^ to obtain sufficient interaction numbers for tracking. Strains: Negative control strain: BBK32-deficient non-infectious adhesion-attenuated B31-A-derived strain (Parent; GCB706: Supplementary Table S1). Positive control strains: Parent expressing *B*. *burgdorferi* vascular adhesin BBK32 (*Bb*-BBK32); BBK32-expressing B31-derived infectious strain (Infectious; GCB726: Supplementary Table S1). *Bb*-Tp0751: Parent expressing Tp0751. Numbers of interactions analyzed/strain: Parent (408), Infectious (647), *Bb*-BBK32 (578), and *Bb*-Tp0751 (288). R^2^ values for linear regressions in (**b**): >0.9. Statistics: one-way ANOVA with Holm-Sidak post-tests (**c**), one-way Kruskal-Wallis ANOVA with Dunn’s post-tests (**d**). *Indicates p < 0.05 vs parent. ^+^Indicates p < 0.05 vs *Bb*-BBK32.

Recently, we developed particle-tracking methods for measuring physical properties of *B*. *burgdorferi*-endothelial interactions under flow, including interaction dissociation rates, velocities, and adhesion complex bond strengths^29^. These methods permit us to determine if specific adhesins stabilize and strengthen interactions by slowing bond dissociation (stabilization) and/or increasing the force that load-bearing adhesion complexes can sustain (strengthening). Using these approaches, we recently reported that BBK32-dependent adhesion complexes have catch bond properties—i.e. become longer-lived with increasing force, and that force-dependent catch bond strengthening begins between 0.5 and 1 dyn/cm^2^, and that BBK32-mediated catch bonds are dependent on pFn^29, 30^. To determine if Tp0751 stabilizes and strengthens endothelial interactions under physiological shear stress, we performed flow chamber experiments at 0.5 and 1 dyn/cm^2^, and measured load-bearing bond dissociation rates, velocities and bond forces for interactions captured by particle-tracking (Fig. 2). Sample time lapse projections of trajectories obtained by particle tracking are shown in Fig. 2a.

We first determined bond dissociation rates of *B*. *burgdorferi*-endothelial interactions for all tracks obtained from 0.5–1 dyn/cm^2^, estimated from the negative slopes of log-transformed frequency distributions of the lifetimes of individual interactions (Fig. 2b,c)^56^. These distributions were linear for all strains (Fig. 2b), indicating that each bacterial-endothelial interaction exhibited single molecule dissociation kinetics—i.e. was formed by a single load-bearing adhesion complex—or was possibly formed by multiple adhesion complexes which dissociated in a synchronous concerted fashion^56^. The dissociation rates for *Bb*-BBK32 and *Bb*-Tp0751 interactions were slower than dissociation rates for the adhesion-attenuated parent (Fig. 2c), indicating that both BBK32 and Tp0751 stabilized interactions. Analysis of strain velocities demonstrated that *Bb*-BBK32 and *Bb*-Tp0751 also moved more slowly over endothelial surfaces than the parent strain (Fig. 2d), indicating that expression of these adhesins slowed interactions. *Bb*-Tp0751 dissociated more slowly but moved faster than *Bb*-BBK32, implying that Tp0751 and BBK32 likely stabilized bacterial-endothelial interactions by different mechanisms. We can conclude from these studies that Tp0751 stabilized and slowed *B*. *burgdorferi*-endothelial interactions under physiological shear stress conditions, but by different mechanisms than BBK32.

### Tp0751-dependent stabilization of tethered and untethered endothelial interactions under shear stress

**Figure 3:**
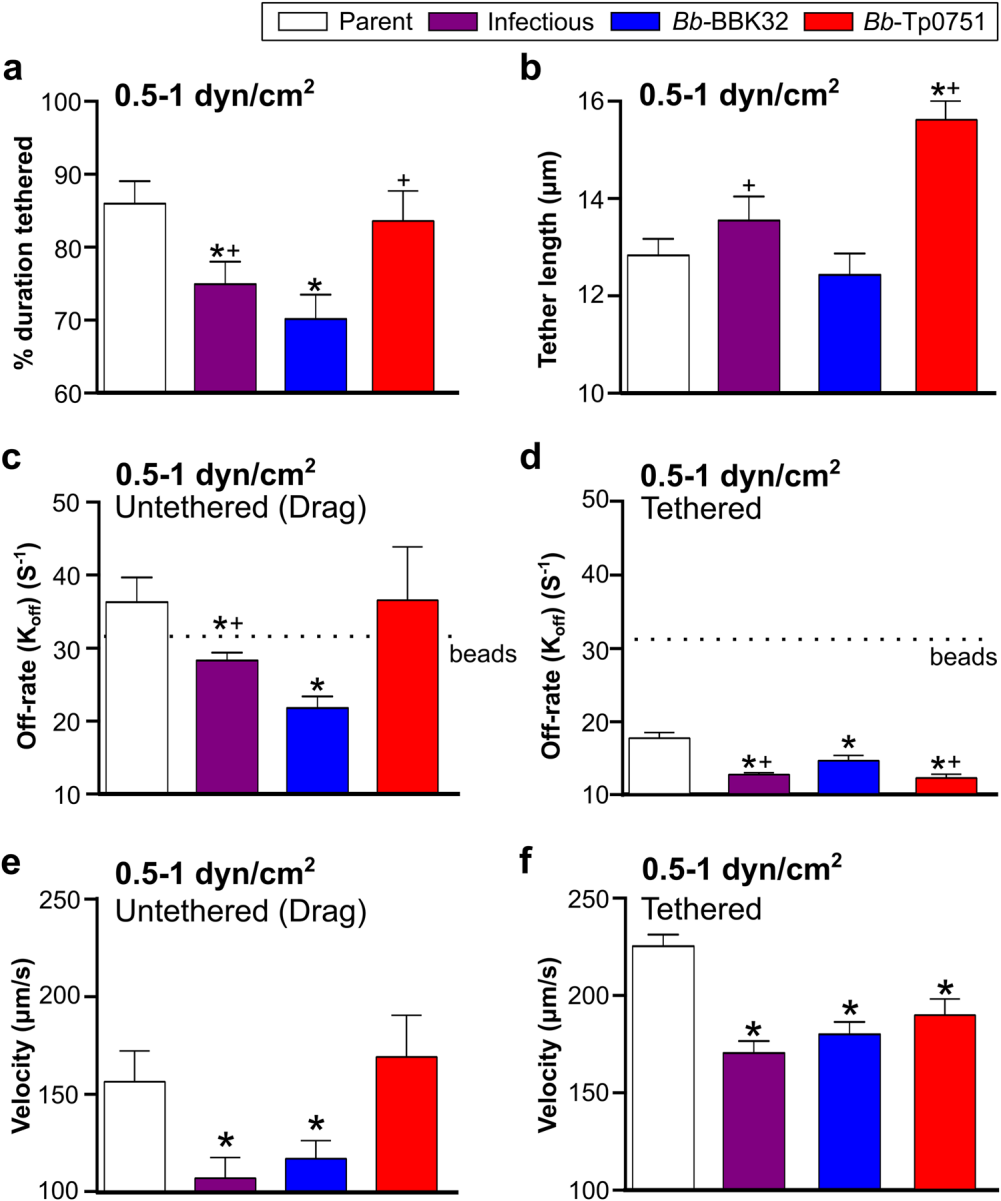
Effect of Tp0751 on tethered and untethered interaction stability and velocity under shear stress. *B*. *burgdorferi* interacts with endothelia under PCV shear stress conditions by two mechanisms: untethered (dragging) interactions, and tethered interactions. Tethered interactions are stabilized by bungee cord-like tethers which anchor bacteria to endothelia, reduce force imposed on load-bearing adhesion complexes and increase adhesion bond stability (lifetime)^29^. (**a**,**b**) Proportion of total bacterial-endothelial interaction times during which bacterial length extended beyond the normal length, indicative of tether formation stabilizing the interaction ((**a**) % duration), and the average tether length (**b**) for all interactions at 0.5–1 dyn/cm^2^. (**c**,**d**) Interaction complex dissociation rates for untethered (**c**) and tethered (**d**) interactions. Dotted lines indicate dissociation rates for non-specific interactions (non-adherent beads). (**e**,**f**) Velocities of untethered (**e**) and tethered (**f**) interactions. Summary values: mean ± 95% CI (**a**,**b**,**e**,**f**), mean ± SEM (**c**,**d**). Interaction properties were calculated from interactions obtained at both 0.5 and 1 dyn/cm^2^. N = 4 and N = 8 independent flow chamber experiments for each strain at 0.5 and 1 dyn/cm^2^, respectively. Strains: Negative control strain: BBK32-deficient non-infectious adhesion-attenuated B31-A-derived strain (Parent; GCB706: Supplementary Table S1). Positive control strains: Parent expressing *B*. *burgdorferi* vascular adhesin BBK32 (*Bb*-BBK32); BBK32-expressing B31-derived infectious strain (Infectious; GCB726: Supplementary Table S1). *Bb*-Tp0751: Parent expressing Tp0751. Numbers of interactions analyzed/strain (**a**,**b**): ≥288; (**c**,**e**): parent (42), infectious (127), *Bb*-BBK32 (130) and *Bb*-Tp0751 (39); (**d**,**f**): parent (366), infectious (520), *Bb*-BBK32 (448), and *Bb*-Tp0751 (249). Statistics: one-way Kruskal-Wallis ANOVA with Dunn’s post-tests (**a**,**b**,**e**,**f**); one-way ANOVA with Holm-Sidak post-tests (**c**,**d**). *Indicates p < 0.05 vs parent. ^+^Indicates p < 0.05 vs *Bb*-BBK32.

The first steps of *B*. *burgdorferi* interactions with PCVs are tethering, where bacteria pause briefly on endothelial surfaces but travel at >100 µm/s (tethered interactions), and dragging, which occurs when bacteria move at <100 µm/s over endothelial surfaces (untethered interactions)^29, 31, 54^. Tethering *B*. *burgdorferi* displace further than their cell length under flow, and are anchored to endothelia by tethers analogous to bungee cords which stabilize interactions; dragging interactions are untethered, and bacteria displace less than their cell length during each adhesion event^29^. Tethering and dragging interactions are mechanistically distinct^29, 55^. Since tethering exerts a strong stabilizing force on interactions^29, 30^, understanding how individual adhesins stabilize interactions also requires examination of adhesin’s effects on untethered interactions, where interpretation of an adhesin’s interaction-stabilizing properties is not confounded by the effects of tethering. BBK32 stabilizes both tethering and dragging interactions^29, 31, 55^. Therefore, we investigated whether Tp0751 also contributed to both of these interaction types (Fig. 3).

*Bb*-BBK32 spent a smaller proportion of the total interaction time tethering compared to the parent strain (Fig. 3a), although the lengths of tethers supporting parent and *Bb*-BBK32 interactions were similar (Fig. 3b). This result implied that the *Bb*-BBK32 strain was likely less reliant on tethering for stabilization of interactions than the parent strain. By contrast, parent and *Bb*-Tp0751 strains spent a similar proportion of the total interaction time tethering (Fig. 3a), and tether lengths were longer for *Bb*-Tp0751 than for both the parent strain and *Bb*-BBK32 (Fig. 3b). This result suggested that *Bb*-Tp0751 was as reliant on tethering for interaction stabilization as the parent strain, but that the tethers associated with Tp0751 adhesion complexes were possibly longer-lived compared to the parent strain and *Bb*-BBK32.

To test these hypotheses, we examined adhesion complex dissociation rates for untethered (dragging) and tethered interactions in *Bb*-Tp0751 and *Bb*-BBK32 strains (Fig. 3c,d). Untethered *Bb*-BBK32 interactions dissociated more slowly than untethered parental interactions (Fig. 3c), demonstrating that BBK32 expression increased the stability (lifetime) of untethered interactions. However, untethered *Bb*-Tp0751 and parental strain interactions dissociated at similar rates, which were comparable to the non-specific dissociation rates of negative control beads (Fig. 3c). Thus, BBK32 stabilized untethered interactions, but Tp0751 did not. In contrast, although both BBK32 and Tp0751 expression increased the stability of tethered interactions compared to the parental strain, tethered *Bb*-Tp0751 interactions dissociated more slowly than tethered *Bb*-BBK32 interactions (Fig. 3d). Thus, Tp0751 promoted endothelial interactions at physiological shear stress specifically by stabilizing tethering.

Finally, we examined how Tp0751 expression affected the velocities of untethered and tethered interactions (Fig. 3e,f). As expected, based on the rapid dissociation rates of untethered Tp0751-dependent interactions (Fig. 3c), *Bb*-Tp0751 bacteria moved as rapidly as the parent strain over endothelial surfaces, whereas *Bb*-BBK32 bacteria moved more slowly than the parent strain (Fig. 3e). The velocities of tethered *Bb*-Tp0751 and *Bb*-BBK32 interactions were slower than the velocity of tethered parental interactions but were similar to each other (Fig. 3f). This result implied that tethers extended at a similar speed in both strains, suggesting that tethers were likely longer in Tp0751-expressing bacteria (Fig. 3b) because they were longer-lived and thus extended further (Fig. 3d). Collectively, these results showed that Tp0751 promoted bacterial association with endothelia under physiological shear stress specifically by stabilizing tethered but not untethered interactions.

### Tp0751 strengthens *B*. *burgdorferi*-endothelial interactions under shear stress

We recently found that under physiological shear stress conditions, BBK32 expression increases the strength of *B*. *burgdorferi*-endothelial interactions, which can be determined by estimating the force imposed on load-bearing adhesion complexes (bond force) during interactions^29^. We also found that BBK32-dependent interactions become longer-lived as bond force increases and thus exhibit catch bond properties^29^. BBK32-dependent catch bond properties are most evident for untethered *B*. *burgdorferi* interactions, where the force sustained by load-bearing bonds is typically greater than for tethered interactions, and is greater than the force threshold at which BBK32-dependent catch bond properties are activated (0.2 pN)^29^.

**Figure 4:**
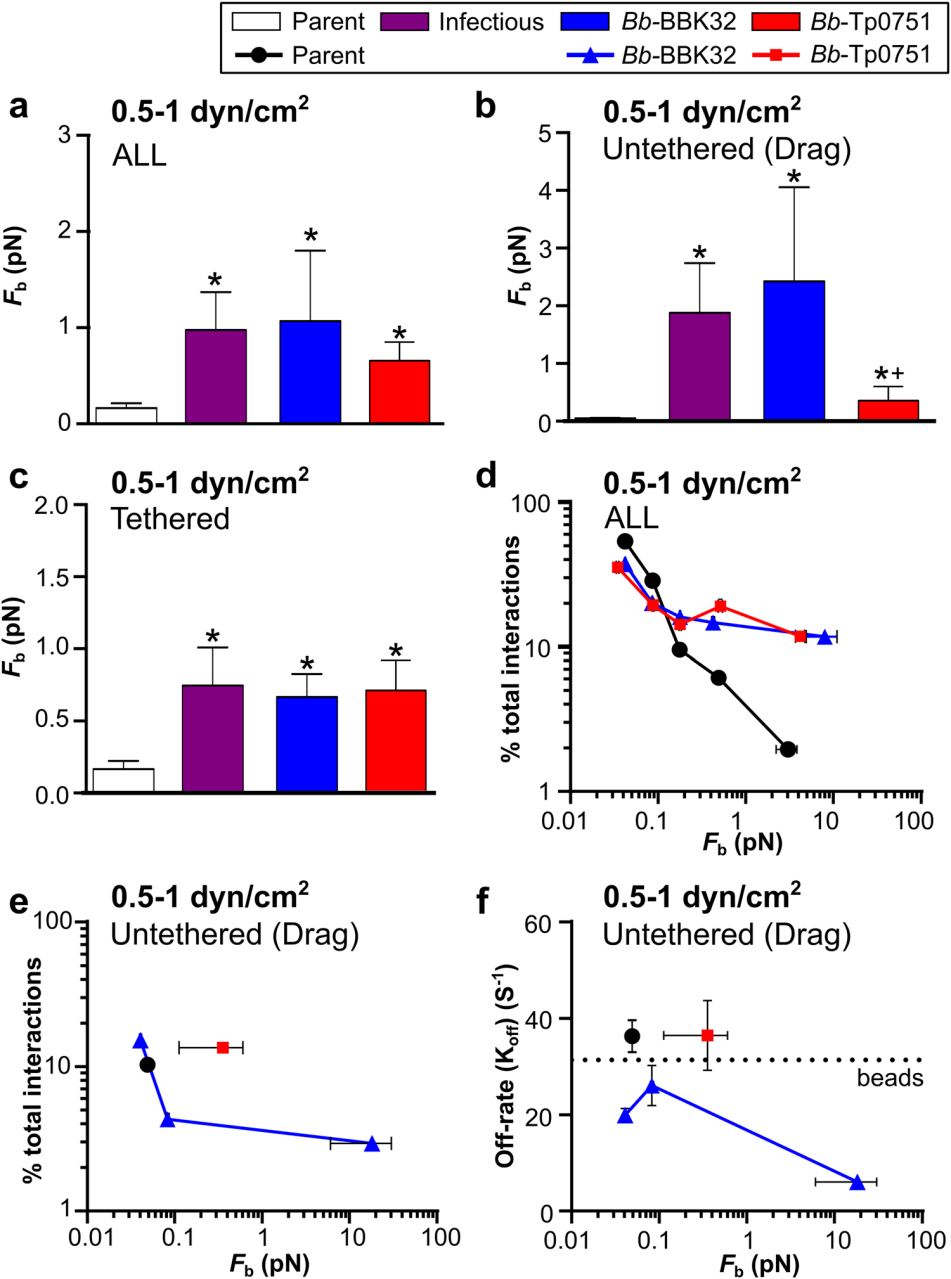
Bond strengths of Tp0751-dependent interactions under shear stress. Forces imposed on individual load-bearing adhesion bonds (*F*_b_) for bacterial-endothelial interactions tracked at 0.5 and 1 dyn/cm^2^ were estimated as described in the Materials and Methods. (**a**–**c**) Bond forces for all (**a**), untethered (**b**) and tethered (**c**) interactions (mean ± 95% CI). (**d**,**e**) Percentages of tracked interactions for each strain occurring at indicated *F*_b_ values. Percentages for both tethered and untethered interactions combined are shown in (**d**), and untethered interactions alone are shown in (**e**). *F*_b_ values for all interactions combined were binned by force range to obtain Mean ± SEM *F*_b_ values plotted on x-axes. For untethered interactions, interactions were grouped by *F*_b_ into a smaller number of bins than for all interactions combined (**e**), to provide sufficient interaction numbers in each bin for accurate calculation of bond dissociation rates at each bond force (**f**). For the parent strain and *Bb*-Tp0751, fewer than 25 interactions were obtained in all but the lowest force bin. For these strains, the data from the higher force bins were therefore pooled with values from the lowest force bin to obtain sufficient interaction numbers for dissociation rate calculations. This resulted in a single data point reflecting the values obtained for all untethered interactions for parent and *Bb*-Tp0751 strains. (**f**) Effect of increasing bond force on mean ± SEM bond dissociation rates. Dotted line indicates the dissociation rate for negative control beads. Strains: Negative control strain: BBK32-deficient non-infectious adhesion-attenuated B31-A-derived strain (Parent; GCB706: Supplementary Table S1). Positive control strains: Parent expressing *B*. *burgdorferi* vascular adhesin BBK32 (*Bb*-BBK32); BBK32-expressing B31-derived infectious strain (Infectious; GCB726: Supplementary Table S1). *Bb*-Tp0751: Parent expressing Tp0751. Numbers of interactions analyzed/strain: ≥288 (**a**,**d**); ≥39 (**b**,**e**,**f**); ≥249 (**c**). Statistics: one-way Kruskal-Wallis ANOVA with Dunn’s post-tests. *Indicates p < 0.05 vs parent. ^+^Indicates p < 0.05 vs BBK32.

To determine if Tp0751 strengthened *B*. *burgdorferi*-endothelial interactions under shear stress, we estimated the forces sustained by load-bearing adhesion complexes during interactions (Fig. 4). Tp0751 expression increased the force sustained by these complexes compared to the parent strain for all interactions (untethered and tethered combined) (Fig. 4a), as well as untethered (Fig. 4b) and tethered (Fig. 4c) interactions. Average *Bb*-Tp0751 and *Bb*-BBK32 bond forces for all interactions were similar (p > 0.05; Fig. 4a), as were bond forces for tethered interactions (p > 0.05; Fig. 4c). However, although untethered *Bb*-Tp0751 interactions sustained greater force than the parent, they were substantially weaker than untethered *Bb*-BBK32 interactions (Fig. 4b). Since untethered *Bb*-Tp0751 interactions were as unstable as untethered parent strain interactions and negative control beads (Fig. 3c), these data implied that Tp0751 expression primarily strengthened tethered interactions.

To estimate the range of forces sustained by Tp0751-dependent adhesion complexes, we binned interactions by bond force, and examined the proportion of total interactions for each strain within each force bin (Fig. 4d,e). For all interaction types combined (untethered and tethered), the proportion of total parent strain interactions declined rapidly at bond forces >0.1 pN. In contrast, the percentage of total *Bb*-Tp0751 and *Bb*-BBK32 interactions remained stable from >0.1 pN up to ~4 and ~8 pN for Tp0751- and BBK32-expressing bacteria, respectively (Fig. 4d). Therefore, expression of Tp0751 and BBK32 increased the upper range of force which could be sustained by load-bearing adhesion complexes by ~40-fold and ~80-fold, respectively.

Untethered BBK32-dependent interactions exhibit catch bond properties above 0.2 pN; in other words, they become longer-lived as bond force increases^29^. To determine if Tp0751 expression conferred catch bond properties, we examined the proportion of total interactions that were untethered as bond force increased, as well as bond dissociation rates (Fig. 4e,f). To obtain sufficient interaction numbers for accurate calculation of dissociation rates, bins containing fewer than 25 interactions were pooled with lower force bins. For the parent strain and *Bb*-Tp0751, all untethered interactions were pooled into a single bin because so few untethered interactions occurred at forces >0.05 pN. The maximum average bond forces that untethered interactions of the parent, *Bb*-Tp0751 and *Bb*-BBK32 strains could sustain were approximately 0.05, 0.35 and 15 pN, respectively (Fig. 4e). Therefore, the force range that untethered Tp0751-dependent interactions could sustain was ~7 times greater than the force range for the parent strain, but ~43 times smaller than the force range for the BBK32 strain. Bond dissociation rates for untethered *Bb*-BBK32 interactions slowed as the force increased above ~0.08 pN, which is consistent with BBK32 conferring catch bond properties to these interactions (Fig. 4f). However, untethered *Bb*-Tp0751 interactions did not become longer-lived as bond force increased (Fig. 4f), indicating that Tp0751 expression did not strengthen interactions by a catch bond mechanism. Collectively, these results indicated that Tp0751 significantly strengthened *B*. *burgdorferi*-endothelial interactions, but over a shorter force range than the *B*. *burgdorferi* catch bond adhesin BBK32.

### Tp0751 promotes interactions with postcapillary venules *in vivo*

**Figure 5:**
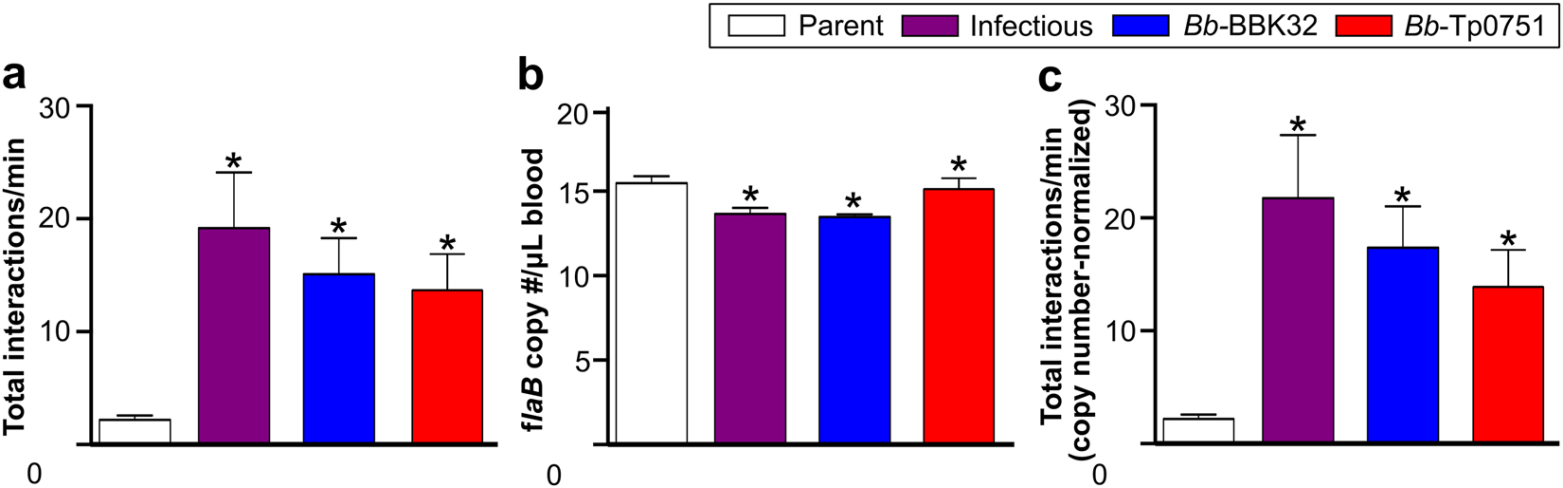
Interaction of Tp0751-expressing *B*. *burgdorferi* with postcapillary venules *in vivo*. (**a**) Total bacterial interactions per minute with dermal PCVs of live mice, visualized by intravital microscopy (IVM). Representative IVM videos are presented in Supplementary Videos S4–S6. Numbers of bacteria that paused and moved more slowly over PCV surfaces under flow were manually counted in time-lapses acquired at 15 fps. (**b**) *B*. *burgdorferi flaB* DNA copy numbers in blood of mice collected after IVM, as measured by quantitative real-time PCR. (**c**) Copy number-adjusted interactions/min in PCVs. Numbers of mice (venules) analyzed per strain: parent: 14 (30); infectious: 8 (22); *Bb*-BBK32: 8 (24); *Bb*-Tp0751: 9 (25). Summary values represent means ± SEM for experiments performed with ≥4 independent bacterial cultures/strain. Statistics: one-way Kruskal-Wallis ANOVA with Dunn’s post-test. *Indicates p < 0.05 vs. parent.

Finally, to determine whether Tp0751-dependent adhesion complexes were sufficiently strong to permit vascular adhesion *in vivo*, we performed intravital microscopy in dermal PCVs of live mice intravenously inoculated with Tp0751-expressing and control *B*. *burgdorferi* strains (Fig. 5), as previously described^31, 54, 55^. Since parent and gain-of-function strains can be cleared more quickly from the circulation than infectious bacteria^55^, microscopy was performed only for the first 20 min after inoculation to reduce differences among experimental groups due to clearance. As shown in Fig. 5a, Tp0751 expression restored vascular interactions to the adhesion-attenuated parent as effectively as BBK32 expression, and both *Bb-*Tp0751 and *Bb-*BBK32 interacted with PCVs as efficiently as the infectious control strain (Fig. 5a). To ensure that Tp0751’s ability to promote interactions was not due to protection of bacteria from clearance compared to the parent strain, we measured copy number of *B*. *burgdorferi flaB* DNA in blood collected from mice immediately after intravital microscopy (IVM) (Fig. 5b), and normalized interaction numbers to bacterial copy number (Fig. 5c). *B*. *burgdorferi* copy number in blood was not greater in any strain compared to the adhesion-attenuated parent strain on the time scale of these experiments (Fig. 5b). Copy number-adjusted interaction numbers for all strains were ~6-, 8- and 10-fold greater than parent strain numbers for *Bb*-Tp0751, *Bb*-BBK32 and infectious bacteria, respectively (Fig. 5c). These results demonstrated that Tp0751 can function as a vascular adhesin *in vivo*.

## Discussion

Like many other invasive pathogens, systemic dissemination via the bloodstream is critical to *T*. *pallidum* pathogenesis. The highly invasive nature of *T*. *pallidum* is shown by the widespread rash characteristic of secondary syphilis, the ability of the bacterium to cross the placental and blood-brain barriers, and the symptomatic involvement of multiple organs and tissues during the course of infection^11, 57^. A critical step in bacterial dissemination via the bloodstream is attachment to the endothelial lining of blood vessels and associated ECM components. Therefore, identifying host-interacting vascular adhesins of *T*. *pallidum* is crucial for advancing our understanding of the pathogenic mechanisms of this organism. The fact that *T*. *pallidum* is an obligate human pathogen that cannot be cultured continuously *in vitro* ^11^ has hindered progress on understanding the molecular interactions involved in the host vasculature-pathogen interface for this organism. Thus, heterologous expression in a related spirochete represents an attractive approach for studying the contributions of *T*. *pallidum* adhesins to these interactions^12, 13^.

A gain-of-function approach such as heterologous expression is a powerful genetic tool that has been successfully used to identify and analyze adhesion-associated phenotypes conferred by pathogenic spirochetes^12, 13, 31, 36, 55, 58–60^. This approach overcomes a major limitation associated with loss-of-function methodologies, where the redundancy of multiple adhesins can mask the binding defect of a single adhesin-deficient mutant. By combining a heterologous gain-of-function approach with flow-chamber, tracking and biophysical analysis methods, which we have recently developed for studying *B*. *burgdorferi*-endothelial interaction mechanisms, it is now feasible to identify and mechanistically characterize the vascular adhesins supporting *T*. *pallidum* dissemination. The ability to study this process in flow chambers, which reproduce the interaction phenotypes observed in the complex environment of PCVs in live mice (Figs 1 and 5)^29^, also means that interaction mechanisms can be systematically dissected by addition, removal and blocking of host components targeted by bacteria expressing known and predicted vascular adhesins. The ability to study bacterial-endothelial interactions in live mice using intravital microscopy also provides a powerful method for testing and validating hypotheses generated in flow chamber studies. Together, these methods have the potential to rapidly advance our understanding of the mechanisms of *T*. *pallidum* dissemination.

The results of this study indicate that, like the *B*. *burgdorferi* adhesin BBK32^29, 31, 55, 59, 61^, Tp0751 can function as a vascular adhesin under static and flow chamber conditions and in PCVs in mice, although the mechanisms of BBK32- and Tp0751-dependent interactions differ (Figs 1, 2, 3, 4 and 5). Tp0751 and BBK32 are multi-functional adhesins that bind to several host ECM components including Fn^37, 42, 46, 47, 49, 61^. Fn plays an important role in *B*. *burgdorferi* tethering and dragging on vascular surfaces^31^, with tethering interactions being facilitated by BBK32 Fn-binding sequences^55^. Our results suggest that Fn may also be important for Tp0751-dependent endothelial interactions (Fig. 1).

Pre-incubation of Tp0751-expressing bacteria (but not endothelia) with pFn inhibited adhesion under static conditions (Fig. 1). Therefore, it is possible that differences in Tp0751- and BBK32-dependent interaction properties under shear stress arise because Tp0751 may target insoluble Fn deposited on endothelial surfaces, whereas BBK32-dependent interactions occur via another mechanism. BBK32 binds to Fn by a tandem β-zipper mechanism involving extensive regions of both proteins^62, 63^ and induces Fn polymerization^64^. By contrast, our recent structural studies reveal that Tp0751 interacts with Fn via positively charged residues on only one face of the protein’s lipocalin fold^49^. Thus, even if Fn is a common component of Tp0751- and BBK32-dependent endothelial interaction mechanisms, it is likely that the affinity of these proteins for Fn and the conformation and physical properties of Fn in adhesion complexes is distinct. Such differences in Fn conformation and/or the mechanisms by which Fn is anchored to surfaces and endothelia might account for the smaller range of force that can be sustained by untethered Tp0751 adhesion complexes (Figs 3 and 4).

Structural and peptide mapping studies also reveal that although Tp0751 forms multiple charge-based contacts with Fn and other ECM proteins, Tp0751 adhesion to endothelia may involve distinct residues on the same surface of the lipocalin fold as the ECM-coordinating residues^49^. These observations predict that the initial tethering association of Tp0751-expressing bacteria with endothelial surfaces may be mediated by charge-based interactions with ECM components such as Fn, but that subsequent dragging adhesion to endothelial cells likely involves a distinct adhesion target. Since untethered dragging interactions were less stable, less abundant and weaker for Tp0751-expressing bacteria than for BBK32-expressing counterparts (Figs 3 and 4), the endothelial targets for dragging interactions may differ for bacteria expressing these adhesins. Binding of ECM components such as Fn to the Tp0751 lipocalin fold may also mask endothelial adhesion sites mediating dragging, suggesting that Tp0751 conformational change or detachment from the ECM could be required to expose endothelial interaction sites, and/or that dragging interactions might be dependent on a second *T*. *pallidum* adhesin which is not present in *B*. *burgdorferi*.

Despite the work that is still required to fully understand the mechanism of Tp0751-dependent endothelial interactions under physiological shear stress, it is nevertheless clear that Tp0751 can function as a vascular adhesin in flow chambers and in dermal PCVs of mice, where shear stress typically ranges from 0.75–1 dyn/cm^2^ ^54^. Our results predict that Tp0751 may be sufficient for bacterial-endothelial interactions in vascular beds of tissues such as the skin where PCV shear stress is typically quite low, but that additional, stronger adhesion mechanisms are likely required for vascular interactions in organs like the brain, where shear stress is typically considerably higher than in the skin. Thus, it is likely that *T*. *pallidum* encodes additional adhesins that collectively permit pathogen interactions with the vasculature of other dissemination sites in the body.

The model systems and biophysical analysis methodologies described herein have successfully identified Tp0751 as a *T*. *pallidum* vascular adhesin. Development of these experimental tools represents a powerful advance that will enable the identification of additional spirochete vascular adhesins and will increase understanding of the molecular mechanisms facilitating vascular attachment of spirochete pathogens, particularly for intractable pathogens such as *T*. *pallidum*.

## Materials and Methods

### Ethics statement

Animal experiments were carried out in accordance with the most recent *Guide to the Care and Use of Experimental Animals* (Canadian Council on Animal Care), and approved by the Animal Care Committee of the University of Toronto (protocol 20010430). Work with *B*. *burgdorferi* was carried out in accordance with University of Toronto, University of Victoria biosafety permits 12a-M30-2 and 2109-107, respectively, and Public Health Agency of Canada and the Canadian Food Inspection Agency guidelines.

### Mice, endothelial cells, *B*. *burgdorferi*

Four to five-week-old male C57BL/6 mice (Charles River Laboratories, Montréal, Canada) were housed at 4 mice/cage, with environmental enrichment and *ad libitum* access to standard chow and water. As described^29^, experiments were conducted with primary human umbilical vein endothelial cells (HUVEC) (Lonza, Allendale, NJ, USA) at passage 2–6. All bacterial strains are listed in the Table S1. GFP-expressing *B*. *burgdorferi* strains were cultivated in selective medium supplemented with 100 μg/ml gentamicin and/or 200 μg/ml kanamycin (Bioshop Canada, Burlington, Canada) as described^54^. All experiments were performed with at least 3 independent cultures/strain. For intravital microscopy experiments, 1% C57BL/6 mouse blood obtained by cardiac puncture was added to cultures 48 hours before imaging^54^.

### Static adhesion assays

As described^12, 65^, HUVEC were grown to confluence in 4-well chamber slides (Nalgene Nunc International, Rochester, NY, USA) coated with 500 μg/mL phenol red-free matrigel (Corning, Tewksbury, MA, USA). GFP-expressing *B*. *burgdorferi* (1.4 × 10^7^) were resuspended in a 3:1 mixture of BSK-II:EGM-2 and added to duplicate wells containing HUVEC. For competitive inhibition experiments, bacteria or HUVEC were pre-incubated with 8 μg of plasma fibronectin (pFn) (Sigma, Oakville, Canada) for 1 hour. Chamber slides were incubated for 12 h, washed with HEPES-buffered saline, and fixed in buffered 10% formalin (Fisher Scientific, Ottawa, Canada). *B*. *burgdorferi* adhered to HUVEC were counted in 10 fields of view/biological replicate using a Nikon 80i fluorescence microscope (Meridian Instrument Company, Kent, WA, USA).

### Flow chamber assays

Flow chamber assays were performed as described^29^. Briefly, HUVEC were cultivated to 2 days post-confluence in Ibidi µ-Slide VI^0.4^ flow chambers (Ibidi GmbH, Planegg, Germany), endothelia were labeled with CellMask Deep Red plasma membrane dye (Life Technologies, Burlington, Canada), and washed *B*. *burgdorferi* diluted to 1 × 10^8^cells/ml in 10% heat-inactivated fetal bovine serum (FBS: Sigma) and Hank’s Balanced Salt Solution (HBSS: Life Technologies) were perfused through flow chambers mounted in a stage-top live cell imaging incubator (Live Cell Instrument, Seoul, Korea) at 0.5 and 1 dyn/cm^2^ (0.284 and 0.568 ml/min, respectively). As reported^29^, live cell imaging was performed at 14–15 frames/second (fps) using a custom-built spinning disk confocal microscope (Quorum Technologies, Guelph, Canada) equipped with a Zeiss 25×/0.8 NA LCI Plan-Apochromat water immersion lens (Carl Zeiss Canada Ltd., Toronto, Canada) and Volocity software v.6.3.0 (Improvision/Perkin-Elmer, Waltham, MA, USA).

### Intravital microscopy (IVM)

IVM was performed in dorsal flank skin as described^55^, with the following exceptions. Eight hundred µl of bacterial suspension (at 2 × 10^9^/ml in PBS) were mixed with 200 µl of 0.1% 2 MDa Texas Red Dextran (Life Technologies) and stored on ice. After warming to room temperature, 250 µl of this mixture were injected via tail vein in mice anesthetized with 10 mg/kg xylazine (MTC Pharmaceuticals, Cambridge, Canada) and 200 mg/kg ketamine hydrochloride (Rogar/STB, Montréal, Canada). Interactions were visualized in dermal postcapillary venules (PCVs) for 5–20 min after injection, using a custom-built high speed multi-wavelength resonant scanner Leica TCS SP8 confocal microscope (Leica, Mannheim, Germany) equipped with argon and DPSS 633 lasers operated at 100% power, a tandem scanner, HCX IRAPO L 25×/0.95 water objective, TD 488/561/633 excitation beam splitters and spectral HyD detectors in photon integration mode (100 gain, 0 offset). Scanning was performed bidirectionally in resonant mode at 8000 Hz over a 1024 × 155 pixel area, at 0.227 µm/pixel and 30 fps with line averaging of 3. Pinhole size was 10.74 AU. Emission wavelengths for green and red channels were 496–550 and 572–795 nm, respectively, and acquisition in red and green channels was simultaneous. Time courses (2 min/PCV) were acquired using Leica Application Suite (LAS) software, and were analyzed post-acquisition after import into Volocity software.

After completion of imaging, blood was collected by cardiac puncture, and qPCR measurement of copies of *B*. *burgdorferi flaB* DNA/µl blood was performed as previously described^55^. Mice were euthanized after cardiac puncture by cervical dislocation.

### Analysis of live cell imaging experiments

As described^29, 54^, *B*. *burgdorferi*-endothelial interactions were manually counted in 100 µm-long straight unbranched regions of PCVs (10–40 µm diameter) and a 30 × 100 µm region of interest (ROI) positioned at the center of flow chamber videos. Interactions were counted as dragging if bacteria took >1 s to pass through this ROI, and were counted as tethering if bacteria passed through the ROI in <1 s but paused as they travelled.

Semi-automated centroid-based tracking of flow chamber videos was performed using a Volocity, custom tracking algorithm as described^29^. Interactions where bacterial area and velocity were >50 µm^2^ and 300 µm/s, respectively, were excluded from analyses. For all analyses except interaction dissociation rates, each interaction was treated as an independent replicate for statistical analysis, since variation in the properties of individual interactions was greater than variation among individual biological replicates^29^.

Interaction dissociation (K_off_) rates were estimated from the negative slopes of linear regressions of log-transformed frequency distributions of the duration of individual interactions in populations^29, 56^. Curve fitting was performed by non-linear regression using the least squares method in GraphPad Prism v.6.02 (GraphPad, La Jolla, CA, USA), with runs post tests. Statistical comparison of the slopes of linear regressions was performed by extra-sum-of-squares F-tests. Dissociation rates for non-specific interactions (beads) are provided in dissociation rate graphs, as calculated previously^29^.

As described^29^, tracked interactions were classified as tethered or untethered based on (a) the ratio of cumulative displacement to bacterial length measured at each time point of each interaction and (b) tethering duration, i.e. the percentage of the total duration of each interaction for which ratios of cumulative displacement to bacterial length at each time point were >1. Untethered interactions (dragging) were those where cumulative displacement was less than bacterial length (ratio <1), and 0% of the total interaction duration was spent tethered. Tethered interactions (tethering) were those where cumulative displacement was greater than bacterial length (ratio >1), and >0% of the total interaction time was spent tethered. Tether length was calculated by dividing the average velocity of each interaction by the dissociation rate calculated for the population. This method corrects for the effect of acquisition frame rate on tether length measurement^29^.

As described^29^, the average force sustained by individual load-bearing adhesion bonds during each interaction (*F*_b_) was calculated by averaging instantaneous *F*_b_ values obtained at each time point within each interaction^29^. The formula used to estimate instantaneous *F*_b_ was *F*_b_ = *F*_s_/cosθ, where θ is the bond angle, *F*_s_ is the force due to flow (31.97τ_w_ *r* ^2^), τ_w_ is wall shear stress and *r* is bacterial projection above the endothelial surface. *r* was calculated from average cell volume values calculated for each bacterial strain as described^29^. This volume differed among strains because of strain-specific differences in average cell length. For experiments reported in this study, average cell volumes for each strain were calculated from average cell lengths for each strain, which were measured under static, no-flow conditions as described^29^. Average cell lengths for GCB706, GCB726, TMB103 and TMB49 were16.34, 16.59, 18.09 and 17.99 µm, respectively).

### Statistical analysis

All statistical analysis was performed in GraphPad Prism. Statistical tests used for all comparisons are described in figure legends and Materials and Methods. Detailed description and justification for statistical methods used to analyze all reported biophysical values (K_off_, *F*_b_, velocity, tether length, % duration tethered) are provided in Ebady *et al*.^29^.

### Data availability statement

Data underlying the findings reported in this manuscript are included in the summarized data presented in the paper and its Supporting Information files. Spreadsheets of raw data can be converted and supplied upon requests directed.

## Electronic supplementary material

Supplementary files

S1 Video

S2 Video

S3 Video

S4 Video

S5 Video

S6 Video
