# Supplementary material for: Identification of Tp0751 (Pallilysin) as a Treponema pallidum Vascular Adhesin by Heterologous Expression in the Lyme disease Spirochete
Source: Sci Rep. 2017 May 8;7:1538. doi: 10.1038/s41598-017-01589-4 (PMC5431505; doi:10.1038/s41598-017-01589-4)
Supplement: Supplementary file 1 — Supplementary files [file 41598_2017_1589_MOESM1_ESM.pdf]

Supporting Information

Identification of Tp0751 (Pallilysin) as a *Treponema pallidum* Vascular Adhesin by Heterologous Expression in the Lyme disease Spirochete

Wei-Chien Andrew Kao[1¶], Helena Pětrošová[1¶], Rhodaba Ebady[1¶], Karen V. Lithgow[2], Pablo Rojas[3], Yang Zhang[1], Yae-Eun Kim[1], Yae-Ram Kim[1], Tanya Odisho[1], Nupur Gupta[1], Annette Moter[4], Caroline E. Cameron[2*] and Tara J. Moriarty[1, 5*]

[1] Matrix Dynamics Group, Faculty of Dentistry, University of Toronto, Toronto, ON, Canada
[2] Department of Biochemistry and Microbiology, University of Victoria, Victoria, BC, Canada
[3] Charité University Medicine Berlin, Berlin, Germany
[4] Biofilmcenter, German Heart Institute Berlin, Berlin, Germany
[5] Department of Laboratory Medicine and Pathobiology, Faculty of Medicine, University of Toronto, Toronto, ON, Canada

[¶]These authors contributed equally to this work.

*Co-corresponding authors: tara.moriarty@utoronto.ca and caroc@uvic.ca. Lead author with primary responsibility for the paper: Tara Moriarty

Supporting Figures

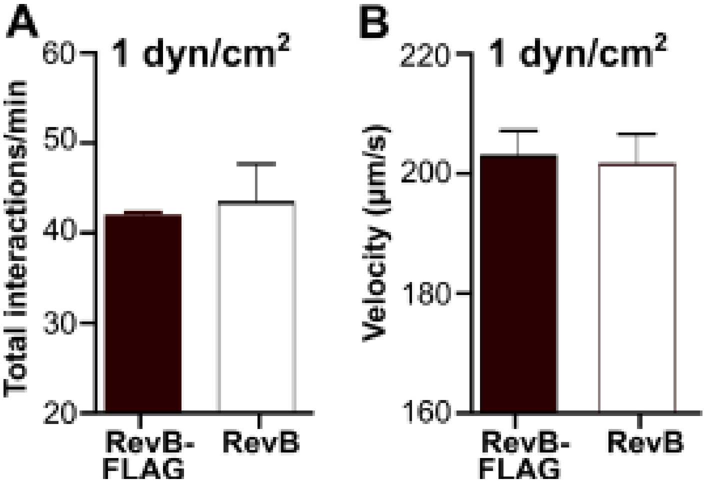

S1 Figure. 3X-FLAG-tag does not affect *B. burgdorferi*-endothelial interactions at 1 dyn/cm^2. (A) Mean ±SEM total interactions/min for non-adherent parental strains expressing C-terminally FLAG-tagged and untagged RevB, an adhesin which does not confer vascular interaction properties to *B. burgdorferi* (Moriarty *et al.*, 2012). Interactions were hand counted. **(B)** Interaction velocities (mean ± 95% CI). Strain names and details are provided in **S1 Table**. N=6 independent biological replicates/strain. Statistics: two-tailed *t*-test (A), two-tailed Mann-Whitney *t*-test (B). $p>0.05$ for all comparisons.

Supporting Tables

S1 Table. *B. burgdorferi* strains used in this study.

Strain number	Background	Description	Antibiotic resistance[a]	No-flow bacterial length of strain (µm) ±SEM	Reference
Parent (GCB706)	B31-A	Non-infectious high passage strain transformed with GFP expression plasmid pTM61	gent	16.34 ± 4.068	Moriarty *et al.* [1]
Infectious (GCB726)	B31 5A4 NP1	Infectious strain transformed with GFP expression plasmid pTM61	gent	16.59 ± 4.982	Moriarty *et al.* [1]
Parent +BBK32/3XFLAG (TMB103)	GCB706	Non-infectious GFP-expressing high passage strain transformed with pTM259 and expressing BBK32-3XFLAG	gent, kan	18.09 ± 3.597	Parker *et al.* [2]
Parent +Tp0751/3XFLAG (TMB49)	GCB706	Non-infectious GFP-expressing high passage strain transformed with pTM259 and expressing Tp0751-3XFLAG	gent, kan	17.99 ± 4.494	Parker *et al.* [2]
Parent +RevB (GCB1586)	GCB706	Non-infectious GFP-expressing high passage strain transformed with pTM255 and expressing RevB	gent, kan	not measured	Moriarty *et al.* [3]
Parent +RevB/3XFLAG (GCB1589)	GCB706	Non-infectious GFP-expressing high passage strain transformed with pTM256 and expressing RevB-3XFLAG	gent, kan	not measured	Moriarty *et al.* [3]

[a]gent – gentamicin, kan – kanamycin.

References

1. Moriarty, T. J. *et al.* Real-time high resolution 3D imaging of the Lyme disease spirochete adhering to and escaping from the vasculature of a living host. *PLoS Pathog.* **4,** e1000090 (2008).
2. Parker, M. L. *et al.* The structure of *Treponema pallidum* Tp0751 (pallilysin) reveals a lipocalin-like fold that mediates adhesion to extracellular matrix components and interactions with host cells. *PLOS Pathog.* **12,** e1005919 (2016).
3. Moriarty, T. J. *et al.* Vascular binding of a pathogen under shear force through mechanistically distinct sequential interactions with host macromolecules. *Mol. Microbiol.* **86,** 1116–1131 (2012).

Supporting Video Captions

S1-S6 Videos. Representative time lapse recordings of *B. burgdorferi*-endothelial interactions in flow chambers and in postcapillary venules of live mice. Two minute timelapse recordings of GFP-expressing *B. burgdorferi* interacting with human endothelial monolayers in flow chambers at 1 dyn/cm^2 (**S1-S3 Videos**) and with dermal postcapillary venules of live mice (**S4-S6 Videos**). **S1, S4 Videos:** parental strain; **S2, S5 Videos**: parental strain expressing BBK32 (*Bb*-BBK32); **S3, S6 Videos**: parental strain expressing Tp0751 (*Bb*-Tp0751). Scale bars: 37 µm (S1-S3 Videos), 25 µm (S3-S6 Videos). Time elapsed is shown in top right corner of videos (total duration of each video: 2 min; frame rate: 15 fps). Endothelial counterstain (flow chambers): live-cell imaging plasma membrane dye (red). Vessel-filling counterstain (postcapillary venules): 2 MDa Texas Red dextran (red).
